# Loss of RIPK3 does not impact MYC-driven lymphomagenesis or chemotherapeutic drug-induced killing of malignant lymphoma cells

**DOI:** 10.1038/s41418-020-0576-2

**Published:** 2020-06-18

**Authors:** Rachel Thijssen, Silvia Alvarez-Diaz, Clea Grace, Ming-yuan Gao, David H. Segal, Zhen Xu, Andreas Strasser, David C. S. Huang

**Affiliations:** 1 The Walter and Eliza Hall Institute of Medical Research, Parkville, VIC 3052 Australia; 2 Department of Medical Biology, University of Melbourne, Parkville, VIC 3050 Australia; 3 Present Address: St Vincent’s Institute of Medical Research, Fitzroy, VIC 3065 Australia

**Keywords:** Cancer genetics, Cell biology

Several studies have shown that loss of expression of the receptor-interacting serine/threonine protein kinase 3 (RIPK3), an essential mediator of necroptotic cell death, promotes the development of cancer, including acute myeloid leukaemia [1]. Moreover, downregulation of RIPK3 has been associated with the resistance of chronic lymphocytic leukaemia cells to necroptotic cell death [1]. Here, we studied the impact of loss of RIPK3 in a mouse model of MYC-driven lymphomagenesis and on the killing of malignant lymphoma cells induced by chemotherapeutics. We found that *Ripk3* deficiency did not accelerate lymphoma development in *Eμ-Myc* transgenic mice. Moreover, the loss of RIPK3 did not alter the sensitivity of *Eμ-Myc* lymphoma cells to chemotherapeutic drugs. These results reveal that RIPK3 does not play critical roles for the development of MYC-driven lymphomas or their therapeutic responses.

To examine the impact of the loss of *Ripk3* on MYC-driven lymphomagenesis, we utilised the *Eμ-Myc* mouse model. *Eμ-Myc;Ripk3*^−/−^ mice displayed a median survival of 97 days (*n* = 80), which was not significantly different from the 118 days seen in control *Eμ-Myc* mice (*n* = 37) (*Eμ-Myc;Ripk3*^−/−^ vs *Eμ-Myc* mice, *p* = 0.0944) (Fig. 1a). At the ethically mandated endpoint, the severity of disease, as determined by the extent of spleen and lymph node enlargement and white blood cell counts, was comparable between mice of the two genotypes (Supplementary Fig. 1a, b). Immuno-phenotyping revealed that all lymphomas in *Eμ-Myc;Ripk3*^−/−^ mice were, as expected, of pro/pre-B (B220^+^sIg^−^) or B (B220^+^sIg^+^) origin, although there was an increased portion of mixed pre-B/B lymphomas compared with the control *Eμ-Myc* mice (Fig. 1b).

**Fig. 1:**
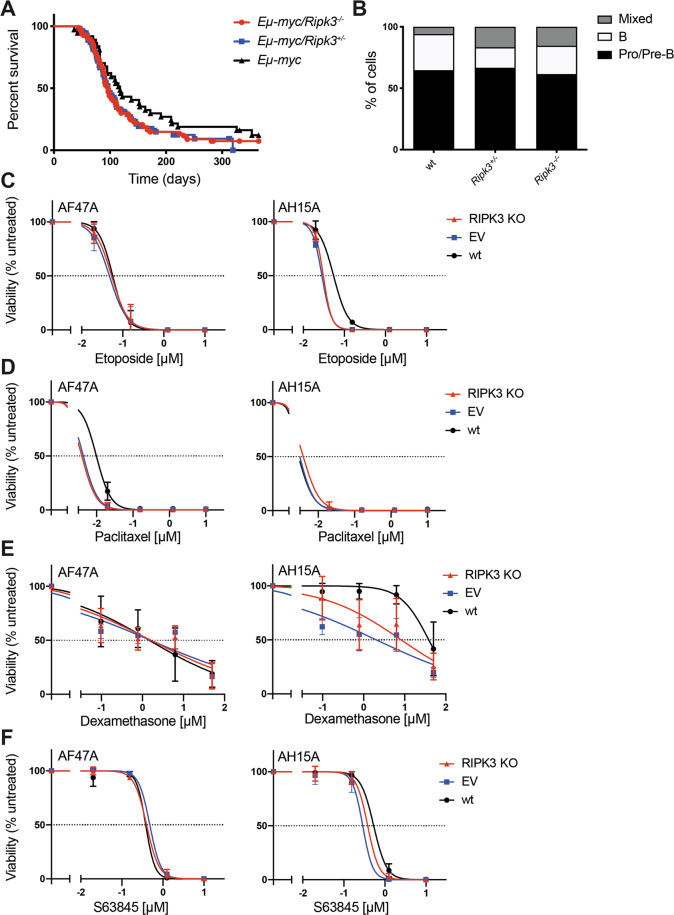
RIPK3 does not play a critical role in the development or therapeutic response in MYC-driven lymphomas. **a** Kaplan–Meier survival curves of *Eμ-Myc* (*n* = 37, median survival of 118 days), *Eμ-Myc;Ripk3*^*+/−*^ (*n* = 54, median survival of 97 days) or *Eμ-Myc;Ripk3*^−/−^ (*n* = 80, median survival of 97 days) mice. The difference in survival between mice of the different the genotypes was not significant (log-rank (Mantel–Cox) test). **b** Stacked bar graph showing the percentages of pro/pre-B (B220^+^sIg^−^), B (B220^+^sIg^+^) and mixed pre-B/B cell lymphomas observed for *Eμ-Myc* (*n* = 34), *Eμ-Myc;Ripk3*^*+/−*^ (*n* = 6) and *Eμ-Myc;Ripk3*^−/−^ (*n* = 13). The difference in surface marker phenotype between lymphomas of the different genotypes was not significant (repeated measures one-way ANOVA, with the Geisser–Greenhouse correction). **c**–**f** *Eμ-Myc* lymphoma cell lines with wt *p53*, AF47A and AH15A, transduced with expression constructs for Cas9 and sgRNAs targeting *Ripk3* (RIPK3 KO) or transduced with an empty vector (EV) were treated with increasing concentrations of etoposide (0–10 μM) (**c**), paclitaxel (0–10 μM) (**d**), dexamethasone (0–50 μM) (**e**) for 48 h, or (**f**) with S63845 (0–10 μM) for 24 h. Cell viability was assessed using Annexin V plus PI staining and FACS analysis. The frequency of Annexin V^−^PI^−^ cells was normalised to control (DMSO treated) cells (Viability (% untreated)). Means ± SD for three independent experiments are shown.

Some other studies have reported that treatment with chemotherapeutic drugs induces the formation of the RIPK1/RIPK3/CASPASE-8 ripoptosome complex that triggers necroptotic cell death and that knockdown of RIPK3 can inhibit this killing of tumour cells [2–6]. To assess whether loss of RIPK3 could affect the response of malignant lymphoma cells to therapeutic agents we made use of CRISPR/Cas9 technology to generate *Eμ-Myc p53* wild-type (AF47A and AH15A) lymphoma cell lines that are deficient for  RIPK3 (Supplementary Fig. 2a, b). The *Ripk3* knockout cells showed similar sensitivity to etoposide, paclitaxel or dexamethasone as the control cells, although some minor differences were observed (Fig. 1c–e). Similarly, the loss of RIPK3 had no impact on the response of lung carcinoma, colon carcinoma or thymic lymphoma derived cell lines to a range of chemotherapeutics [7, 8]. Human and mouse tumour cells driven by deregulated over-expression of c-MYC are highly dependent on the anti-apoptotic protein MCL-1 for survival and the MCL-1-specific BH3 mimetic drug S63845 induces dose dependent apoptotic death in murine *Eμ-Myc* as well as human Burkitt’s lymphoma cell lines [9, 10]. Loss of RIPK3 did not affect the response of *Eμ-Myc* lymphoma cell lines to S63845 (Fig. 1f). Staining with Annexin V and propidium iodide (PI) revealed that both the parental and the Ripk3-deleted *Eμ-Myc* lymphoma cells showed classical features of apoptosis (Annexin V^+^PI^−^) at early time points after treatment with chemotherapeutic drugs (Supplementary Fig. 3). Accordingly, loss of the pro-apoptotic effectors BAX and BAK rendered the *Eμ-Myc* lymphoma cells completely resistant to these agents (Supplementary Fig. 3).

Collectively, these findings demonstrate that RIPK3, and by extension necroptotic cell death, is not a major suppressor of MYC-driven lymphoma development. Moreover, the results indicate that RIPK3 and necroptosis do not play major roles in the response of malignant lymphoma cells to a range of anti-cancer agents, at least under tissue culture conditions. As the *Eμ-Myc* mice constitute a model of human Burkitt’s lymphoma, it therefore remains possible that RIPK3 and necroptosis may play a role in other types of lymphoma or other malignancies. Moreover, RIPK3 and necroptosis might play a role in the response of *Eμ-Myc* lymphomas (and other tumours) to anti-cancer agents in vivo, perhaps by impacting anti-tumour immune responses from the host, and we will make our cell lines available for such investigations.

## Supplementary information

Supplementary information for RIPK3 is not essential for MYC-driven lymphomagenesis and chemotherapeutic drug-induced killing of malignant lymphoma cells
