# Supplementary material for: Loss of RIPK3 does not impact MYC-driven lymphomagenesis or chemotherapeutic drug-induced killing of malignant lymphoma cells
Source: Cell Death Differ. 2020 Jun 18;27(8):2531–3. doi: 10.1038/s41418-020-0576-2 (PMC7370228; doi:10.1038/s41418-020-0576-2)
Supplement: Supplementary file 1 — Supplementary information for RIPK3 is not essential for MYC-driven lymphomagenesis and chemotherapeutic drug-induced killing of malignant lymphoma cells [file 41418_2020_576_MOESM1_ESM.pdf]

Supplementary information for RIPK3 is not essential for MYC-driven lymphomagenesis and chemotherapeutic drug-induced killing of malignant lymphoma cells

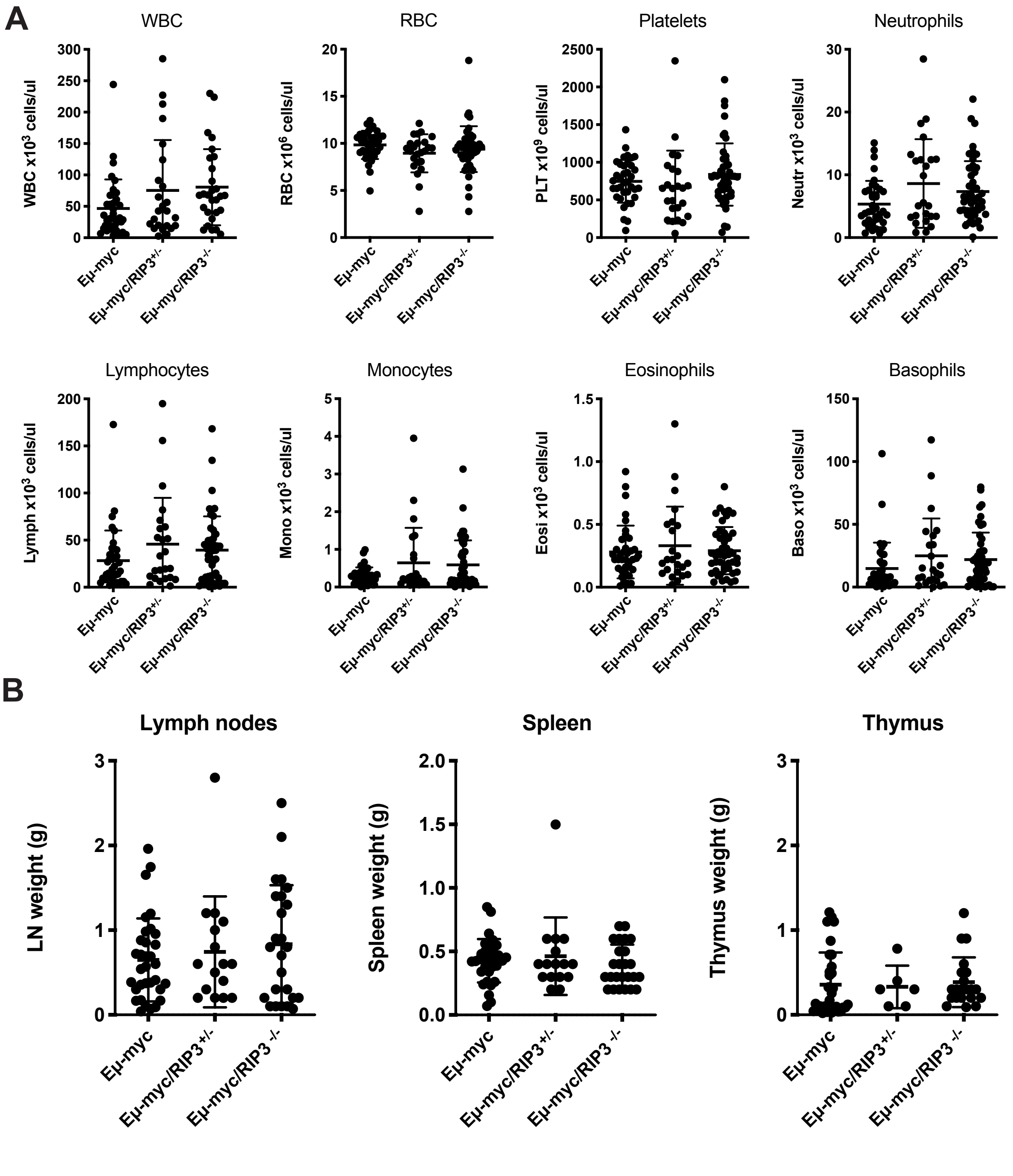

Supplemental Figure 1. Ripk3 loss does not affect white blood cell counts or spleen and lymph node enlargement in *Eμ-Myc* transgenic mice. A-B. Scatter plots of blood cell counts (white blood cells; WBC, red blood cells; RBC, platelets, neutrophils, lymphocytes, monocytes, eosinophils, basophils) **(A)** and weights of lymph nodes (inguinal, axillary and brachial lymph nodes), spleen and thymus **(B)** of sick *Eμ-Myc*, *Eμ-Myc;Ripk3*$^{+/-}$ and

Eμ-Myc;Ripk3$^{-/-}$ mice that had reached ethical endpoint. Bars represent mean ± SD. No significant differences were found (one-way ANOVA (Tukey's multiple comparisons test)).

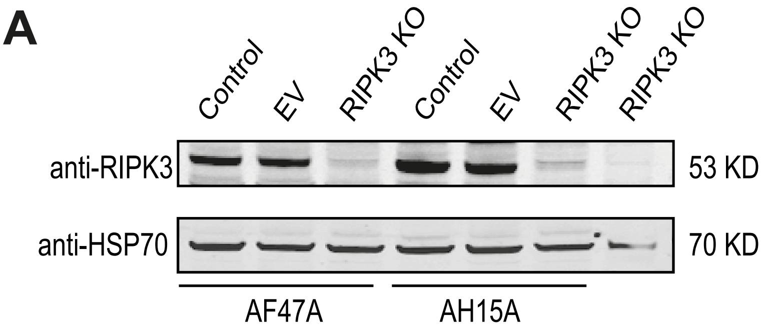

B

AF47A infected with sgRIP3

		sgRIP3	Reads #
**WT**	5'-..	GACTCTGGGGGCACCCTAG--CGTACTTGGACCCAGAGCTGTTATTTGATGTCAACC ..-3'	
**D97**		GACTCTGGGGGCACCCTAG--AG------------------------------	301555
**D20**		GACTCTG--------------------ACCCAGAGCTGTTATTTGATGTCAACC	11198
**Native**		GACTCTGGGGGCACCCTAG--CGTACTTGGACCCAGAGCTGTTATTTGATGTCAACC	902
**+1**		GACTCTGGGGGCACCCTAG-CCGTAC TTGGACCCAGAGCTGTTATTTGATGTCAACC	563
**+2**		GACTCTGGGGGCACCCTAGCCCGTACTTGGACCCAGAGCTGTTATTTGATGTCAACC	418
**D33**		---------------------------ACCCAGAGCTGTTATTTGATGTCAACC	408
**D2**		GACTCTGGGGGCACCCTAG--A--GCTTGGACCCAGAGCTGTTATTTGATGTCAACC	243
**D16**		GACTCTGGGGGCACC----------------CAGAGCTGTTATTTGATGTCAACC	104
**D60**		--TTGATGTCAACC	103
**D12**		GACTCTGGG--------------TACTTGGACCCAGAGCTGTTATTTGATGTCAACC	83

% indel = 98%

AH15A infected with sgRIP3

		sgRIP3	Reads #
**WT**	5'-..	GACTCTGGGGGCACCCTAG-- CGTACTTGGACCCAGAGCTGTTATTTGATGTCAACC ..-3'	
**Native**		GACTCTGGGGGCACCCTAG-- CGTACTTGGACCCAGAGCTGTTATTTGATGTCAACC	4267
**+2**		GACTCTGGGGGCACCCTAGGGCGTACTTGGACCCAGAGCTGTTATTTGATGTCAACC	3481
**+1**		GACTCTGGGGGCACCCTAG- CCGTAC TTGGACCCAGAGCTGTTATTTGATGTCAACC	2447
**+1**		GACTCTGGGGGCACCCTAG- TCGTACTTGGACCCAGAGCTGTTATTTGATGTCAACC	1805
**+1**		GACTCTGGGGGCACCCTAG- GCGTACTTGGACCCAGAGCTGTTATTTGATGTCAACC	1095
**+1**		GACTCTGGGGGCACCCTAG- ACGTACTTGGACCCAGAGCTGTTATTTGATGTCAACC	884
**+2**		GACTCTGGGGGCACCCTAGCTCGTACTTGGACCCAGAGCTGTTATTTGATGTCAACC	810
**D16**		GACTCTGGGGGCACC----------------CAGAGCTGTTATTTGATGTCAACC	685
**+2**		GACTCTGGGGGCACCCTAGACCGTACTTGGACCCAGAGCTGTTATTTGATGTCAACC	615
**D19**		GACTCT-------------------TGGACCCAGAGCTGTTATTTGATGTCAACC	563

% indel = 74.4%

Supplemental Figure 2. Generation of *Eμ–Myc* p53 wild-type AF47A and AH15A lymphoma cell lines that are deficient for Ripk3.

A. Western analysis for Ripk3 in lysates from pools of the *Eμ–Myc* lymphoma cell lines AF47A and AH15A that had been transduced with vectors for Cas9 and sgRNAs targeting *Ripk3* or a control sgRNA. As a control, we used the lysate prepared from E14 foetal livers from a *Ripk3*$^{-/-}$ mouse.

B. Genomic DNA prepared from AF47A and AH15A *Eμ–Myc* lymphoma cells expressing an sgRNA targeting *Ripk3* were subjected to Sanger DNA sequencing. The table shows the top 10 sequencing reads. Grey dashes: deleted bases, grey letters: insertions or substitutions.

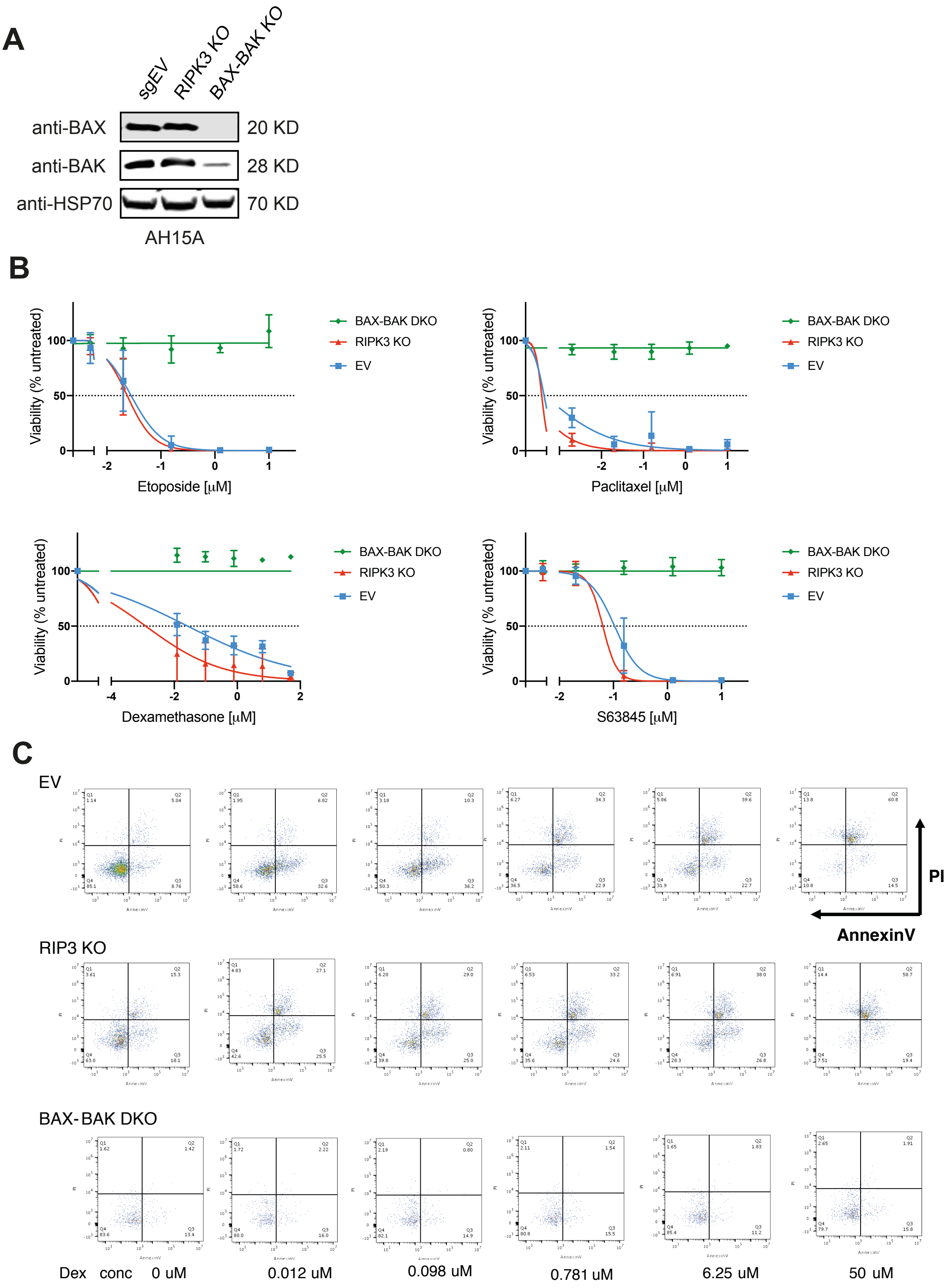

Supplemental Figure 3. Anti-cancer agents induce cell death via apoptosis in *Eμ-Myc* lymphoma cells.

A. Western blot analysis for Bax and Bak in lysates from pools of the *Eμ–Myc* lymphoma cell line AH15A that had been transduced with vectors for CAS9 and sgRNAs targeting *Bax* and *Bak* or a sgRNA targeting *Ripk3*, or a control sgRNA (EV).

B. *Eμ–Myc* lymphoma cell line AH15A, transduced with expression constructs for Cas9 and sgRNAs targeting *Bax* and *Bak* (Bax/Bak double knock-out (DKO), or a sgRNA targeting *Ripk3* (Ripk3 KO) or transduced with an empty vector (EV) were treated with increasing concentrations of etoposide (0-10 μM), paclitaxel (0-10 μM) or dexamethasone (0-50 μM) for 48 h or were treated with S63845 (0-10 μM) for 24 h. Cell viability was assessed using AnnexinV plus PI staining and FACS analysis. AnnexinV$^-$PI$^-$ cells are normalised to the control (DMSO treated) cells (Viability (% untreated)). Means ± SD for three independent experiments are shown.

C. Flow cytometry plots of AnnexinV (X-axis) and PI (Y-axis) staining of empty vector (EV), Ripk3 KO or Bax/Bak DKO AH15A *Eμ–Myc* lymphoma cells treated with increasing concentrations of dexamethasone.

Methods

Experimental animals: All experiments with mice were performed according to the guidelines of The Walter and Eliza Hall Institute of Medical Research Animal Ethics Committee. The *Eμ-Myc* transgenic mice expressing the *c-Myc* oncogene under the control of the immunoglobulin heavy chain gene enhancer (*Eμ*) (1, 2) have been backcrossed with C57BL/6 mice for >20 generations. The *Ripk3*$^{-/-}$ mice (on a C57BL/6 background) have been described (3). *Eμ-Myc* transgenic males were crossed with *Ripk3*$^{-/-}$ females to generate *Eμ-Myc;Ripk3*$^{-/-}$ offsprings.

***Eμ-Myc* lymphomas and *Eμ-Myc* lymphoma cell lines:** *Eμ-Myc* lymphomas were characterised by flow cytometry as either pro-B/pre-B (B220^{+} sIg^{-}) or B cell lymphomas (B220^{+} sIg^{-}). Lymphoma cells were passaged *in vitro* to obtain stably growing lymphoma cell lines. Lymphoma cell lines were cultured at 37°C in a humidified 10% CO_2 incubator in high-glucose Dulbecco's modified Eagle's medium supplemented with 10% foetal calf serum (SAFC Biosciences, Lenexa, KS, USA), 50 μM 2-mercaptoethanol (Sigma) and 100 mM asparagine (Sigma), hereafter referred to as FMA.

CRISPR/Cas9-mediated genome editing: The *Eμ-Myc* lymphoma cell lines AF47A and AH15A, both wild-type for p53, were serially infected with lentiviruses that stably express CAS9 with the fluorescent marker mCherry or the sgRNA vectors listed below. A sgRNA targeting *Ripk3* was cloned into FgH1tUTG and sgRNAs targeting *Bax* or *Bak* were cloned into pKLV2-U6gRNA5(BbsI)-PGKpuro2ABFP-W. As a control, AF47A and AH15A cells expressing CAS9 were transduced with FgH1tUTG empty vector (EV). The sequence of the sgRNA targeting *Ripk3* used in this study is 5'-TAGCGTACTTGGACCCAGA-3'. The sequence of the sgRNA targeting *Bax* is 5'- GCGAATTGGAGATGAACTGC-3' and the one for targeting *Bak* is 5'- CTGCCAACCCCGAGATGGAC-3'. Double positive cells (mCherry^{+}GFP^{+} or mCherry^{+}BFP^{+}) were sorted using a BD FACSAria W Sorter (BD Biosciences). All experiments were undertaken with pools of infected cells and gene knock-out was verified by Western blotting and DNA sequencing. Sequences of primers for targeted PCR used in this study are 5'-*GTGACCTATGAACTCAGGAGTC*GCAGATTTTGGCCTGTCCAC-3' and 5'-*CTGAGACTTGCACATCGCAGC*GGGTTTAGGTGCTTTCGGGT-3'. The overhang sequences (in italic) at the 5' ends of the unique *Ripk3* primers are designed to aid rapid sequencing of the PCR products that are designed to span the region targeted by the sgRNA.

Cell death assays: *Eμ-Myc* lymphoma cell lines AF47A and AH15A, both wild-type for p53, either control (EV) and *Ripk3* KO were seeded in flat-bottomed 96-well plates at 2×10^4 cells per well. 6-point 1:8 serial dilutions of etoposide (Hospira, Mulgrave, VIC, Australia), paclitaxel (#T7191, Sigma) S63845 (#A-6044, Active Biochem, Wanchai, Hongkong) starting from 10 μM or dexamethasone (#P4902, Sigma) starting from 50 μM were used for the tests. After 48 h or 24 h for S63845, cells were stained with Annexin V-APC (WEHI) and propidium iodide (PI, #P4864, Merck), and cell viability was assessed by PI and Annexin V exclusion using a FACSCanto (BD). FACS data were analysed using Flowjo software.

Western blotting: Protein samples were size-fractionated by SDS-PAGE and then blotted onto nitrocellulose (Invitrogen, Grand Island, NY, USA) membranes. Membranes were blocked with 5% non-fat dry milk (Devondale, Melbourne, VIC, Australia) in PBS with 0.1% Tween 20 (Sigma) and then probed with antibodies against RIPK3 (ProSci, Poway, CA, USA), BAK (B5897 Sigma), BAX (49F9, WEHI) and HSP70 (N6; (gift from Dr Robyn Andersson, Peter MacCallum Cancer Centre, Melbourne, Australia). Detection was performed with IR800-conjugated goat anti-mouse IgG or Alexa Fluor 680-conjugated goat anti-rabbit IgG or rat IgG antibodies (Rockland, Limerick, PA, USA) and signals were detected by Odyssey Imaging System (Li-Cor, Lincoln, NE, USA). Lysates from tissues of a *Ripk3*$^{-/-}$ mouse (4) was used as a control.

Statistical analysis: Prism (GraphPad Prism, GraphPad Software Inc., La Jolla, CA, USA) software was used for statistical analysis. Multi-group comparisons of leukaemic burden, lymphocyte counts or the weights of lymph nodes, spleen and thymus used one-way ANOVA using Tukey's multiple comparisons test. Mouse survival data were plotted using Kaplan–Meier curves. Differences in survival time to tumour-related deaths of *Eμ-Myc* mice of the different genotypes were tested using log-rank tests (each independent lymphoma analysed separately). p values <0.05 were considered to indicate statistical significance.

Supplementary references

1. Adams JM, Harris AW, Pinkert CA, Corcoran LM, Alexander WS, Cory S, et al. The c-myc oncogene driven by immunoglobulin enhancers induces lymphoid malignancy in transgenic mice. Nature. 1985;318(6046):533-8.
2. Harris AW, Pinkert CA, Crawford M, Langdon WY, Brinster RL, Adams JM. The E mu-myc transgenic mouse. A model for high-incidence spontaneous lymphoma and leukemia of early B cells. J Exp Med. 1988;167(2):353-71.
3. Newton K, Sun X, Dixit VM. Kinase RIP3 is dispensable for normal NF-kappa Bs, signaling by the B-cell and T-cell receptors, tumor necrosis factor receptor 1, and Toll-like receptors 2 and 4. Mol Cell Biol. 2004;24(4):1464-9.
4. Brumatti G, Ma C, Lalaoui N, Nguyen NY, Navarro M, Tanzer MC, et al. The caspase-8 inhibitor emricasan combines with the SMAC mimetic birinapant to induce necroptosis and treat acute myeloid leukemia. Sci Transl Med. 2016;8(339):339ra69.

Author contributions

DCSH and AS designed the research; RT, SAD, MYG, CG, DHS and ZX performed experiments; RT and SAD analysed data; RT, SAD, AS and DCSH wrote the paper. All authors read and approved the final manuscript.
